# Establishment and analysis of a disease risk prediction model for the systemic lupus erythematosus with random forest

**DOI:** 10.3389/fimmu.2022.1025688

**Published:** 2022-11-01

**Authors:** Huajian Chen, Li Huang, Xinyue Jiang, Yue Wang, Yan Bian, Shumei Ma, Xiaodong Liu

**Affiliations:** ^1^ School of Public Health and Management, Wenzhou Medical University, Wenzhou, China; ^2^ South Zhejiang Institute of Radiation Medicine and Nuclear Technology, Wenzhou Medical University, Wenzhou, China; ^3^ Key Laboratory of Watershed Science and Health of Zhejiang Province, Wenzhou Medical University, Wenzhou, China

**Keywords:** systemic lupus erythematosus, Lasso, random forest, GEO, disease risk prediction model

## Abstract

Systemic lupus erythematosus (SLE) is a latent, insidious autoimmune disease, and with the development of gene sequencing in recent years, our study aims to develop a gene-based predictive model to explore the identification of SLE at the genetic level. First, gene expression datasets of SLE whole blood samples were collected from the Gene Expression Omnibus (GEO) database. After the datasets were merged, they were divided into training and validation datasets in the ratio of 7:3, where the SLE samples and healthy samples of the training dataset were 334 and 71, respectively, and the SLE samples and healthy samples of the validation dataset were 143 and 30, respectively. The training dataset was used to build the disease risk prediction model, and the validation dataset was used to verify the model identification ability. We first analyzed differentially expressed genes (DEGs) and then used Lasso and random forest (RF) to screen out six key genes (OAS3, USP18, RTP4, SPATS2L, IFI27 and OAS1), which are essential to distinguish SLE from healthy samples. With six key genes incorporated and five iterations of 10-fold cross-validation performed into the RF model, we finally determined the RF model with optimal mtry. The mean values of area under the curve (AUC) and accuracy of the models were over 0.95. The validation dataset was then used to evaluate the AUC performance and our model had an AUC of 0.948. An external validation dataset (GSE99967) with an AUC of 0.810, an accuracy of 0.836, and a sensitivity of 0.921 was used to assess the model’s performance. The external validation dataset (GSE185047) of all SLE patients yielded an SLE sensitivity of up to 0.954. The final high-throughput RF model had a mean value of AUC over 0.9, again showing good results. In conclusion, we identified key genetic biomarkers and successfully developed a novel disease risk prediction model for SLE that can be used as a new SLE disease risk prediction aid and contribute to the identification of SLE.

## Highlights

1. Multiple GEO datasets were merged into one large sample data.
2. Analysis of SLE biomarkers using whole blood.
3. The Lasso and random forest joint exploration of SLE biomarkers.
4. For the first time, a robust random forest algorithm-based SLE disease risk prediction model was developed using 6 key genes (OAS3, USP18, RTP4, SPATS2L, IFI27 and OAS1).
5. Models are not only constructed in arrays, but also built in the context of high-throughput sequencing.

## Introduction

Systemic lupus erythematosus (SLE) is an autoimmune disease characterized by multi-organ inflammatory damage and widespread autoantibodies, mainly affecting women of childbearing age (1). The heterogeneity of the clinical manifestations of SLE can affect multiple organs including the skin, joints, central nervous system, vascular system and kidneys (2). Dysregulation of the immune system is one of the main causes of the pathogenesis of SLE, in which abnormal activation of B and T cells leads to loss of immune tolerance to autoantigens as well as to a high frequency of autoantibody production in lupus (1, 3). SLE is a disease that cannot be cured (4) and requires lifelong medication. Its pathogenesis is still not fully understood, and its effective identification becomes a crucial role, as it can improve patient prognosis. Due to the key role that immune dysregulation plays in SLE, immune biomarkers have emerged to help better diagnose SLE and thus improve disease control (5). However, the variety and non-specific symptoms of SLE make it difficult to obtain a correct and timely diagnosis (6). Therefore, there is an urgent need for more precise diagnostic and therapeutic targets for SLE. Over the past decade or so, rapid advances in microarray and high-throughput sequencing technologies have provided a reliable and extensive approach to deciphering the genetic and epigenetic landscapes of disease. The wealth of evidence provided simultaneously facilitates the prediction of various diseases (7, 8). Vilhjálmsson et al. reported that prediction models based on multiple biomarkers can significantly improve predictive accuracy (9). However, feature selection remains a major bottleneck in building multi-gene classification models. This concern is well addresses by the application of various machine learning techniques in biology nowadays (10–12). These algorithms, when used individually or in combination, have made significant contributions to the classification of gene expression data, disease detection, and microbiome studies (13–15).

We developed a novel disease risk prediction model for SLE at the transcriptome level based on the key genes screened in the GEO database. Lasso and RF were first used to jointly determine which genes were most important for SLE classification. Then, the RF of optimal mtry was selected by grid search, and a genetic disease risk prediction model for SLE was developed based on the key genes. We evaluated the performance of the disease risk prediction model using a validation dataset to confirm its accuracy and discriminatory power. In addition, we performed the disease risk prediction model for SLE modeling not only on array but also on high-throughput sequencing as well.

## Materials and methods

### Data sources

The datasets for this study were obtained from the Gene Expression Omnibus (GEO) database, a gene expression database created and maintained by the National Center for Biotechnology Information (NCBI) (https://www.ncbi.nlm.nih.gov/geo/query/acc.cgi). The study was conducted using the keyword “systemic lupus erythematosus”, which was extensively searched through the NCBI database platform. The type of dataset we chose was array expression profiling and high-throughput sequencing, the type of organism was Homo sapiens, the sample type was whole blood, and the sample size of the dataset was greater than 30.

The datasets GSE138458, GSE154851, GSE50635, GSE61635, GSE99967, GSE185047, GSE72509, GSE110685 and GSE112087 were obtained. GSE138458 is a dataset containing 307 SLE patient samples and 23 healthy samples. The whole blood samples were obtained from the Oklahoma Medical Research Foundation. The gene expression was analyzed using the Illumina HumanHT-12 V4.0 expression bead chip. GSE154851 is a dataset containing 38 SLE patient samples and 32 healthy samples. The whole blood samples were obtained from Trakya University. The gene expression was analyzed using Agilent-039494 SurePrint G3 Human GE v2 8x60K Microarray 039381 (Feature Number version). GSE50635 is a dataset containing 33 SLE patient samples and 16 healthy samples. The whole blood samples were obtained from Mayo Clinic. The gene expression was analyzed using the Affymetrix Human Gene 1.0 ST Array [transcript (gene) version]. GSE61635 is a dataset containing 99 SLE patient samples and 30 healthy samples. The whole blood samples were obtained from Eli Lilly and Company. Gene expression was analyzed using the Affymetrix Human Genome U133 Plus 2.0 Array. GSE110685 is a dataset containing 36 SLE patient samples and 18 healthy samples. The whole blood samples were obtained from NIAMS. Gene expression was analyzed using the Illumina HiSeq 2500 (Homo sapiens). GSE112087 is a dataset containing 62 SLE patient samples and 59 healthy samples. The whole blood samples were obtained from CSL Limited/bio21 Institute. Gene expression was analyzed using the Illumina HiSeq 2500 (Homo sapiens). GSE112087 is a dataset containing 99 SLE patient samples and 19 healthy samples. The whole blood samples were obtained from Genentech. Gene expression was analyzed using the Illumina HiSeq 2500 (Homo sapiens). The information about the seven datasets and the two external validation datasets (GSE99967 and GSE185047) is displayed in **Table 1** and **Table S1**.

**Table 1:** The information on the Systemic lupus erythematosus (SLE) datasets of the gene expression omnibus (GEO).

GEO accession	Expression profiling	Tissue	SLE	Health	Total
GSE138458	Array	Whole blood	307	23	330
GSE154851	Array	Whole blood	38	32	70
GSE50635	Array	Whole blood	33	16	49
GSE61635	Array	Whole blood	99	30	129
GSE99967	Array	Whole blood	38	17	55
GSE185047	Array	Whole blood	87	0	87
GSE110685	High-throughput sequencing	Whole blood	36	17	53
GSE112087	High-throughput sequencing	Whole blood	62	58	120
GSE72509	High-throughput sequencing	Whole blood	99	18	117

### Data processing

Next, we corrected the quantile-normalized signal intensity for the log2-transformed dataset of the array expression spectrum and output the correction results. The dataset of high-throughput sequencing only output the results after log2 change, in which we transformed the Count of GSE112087 into RPKM and output the results after log2 change to maintain consistency with the data form of GSE72509 and GSE110685. Finally, we merged the datasets of array expression spectra with those of high-throughput sequencing separately each and used the ComBat function in the sva package to remove the batch effect of data from different platforms (**Supplement Figure S1A**).

### Stratified random sampling

To be able to better represent the robustness of the disease risk prediction model, we use stratified random sampling method for reasonable sample division. We used the createDataPartition function in the R package caret to divide the datasets (GSE138458, GSE154851, GSE50635, and GSE61635) of the array expression spectrum into a training dataset and a validation dataset, with a sample size ratio of 7:3. The training dataset is used to develop the disease risk prediction model and the validation dataset to verify the effectiveness of the model.

### Screening for DEGs

Differential expression analysis was performed using traditional Bayesian methods to screen the training dataset for DEGs using the limma package. A false discovery rate (FDR) less than 0.05 and an absolute value of log2 fold change (log2FC) greater than 1 were used as significance criteria for DEGs. DEGs heatmap was created using the pheatmap package. The volcano map was created using the ggplot2 package.

### Gene enrichment analysis

The DEGs were interpreted using Gene Ontology (GO), Kyoto Encyclopedia of Genes and Genomes (KEGG) and Disease Ontology (DO). In this study, we used the clusterProfiler package for GO and KEGG analysis, and the DOSE package for DO analysis. The GO was interpreted in terms of biological processes (BP). We have shown them in the form of a ring diagram. In addition, we again performed the Gene Set Enrichment Analysis (GSEA) study using the clusterProfiler package to pinpoint the pathway differences between SLE and normal blood. As SLE has historically been studied in relation to the immune system, we immediately investigated the potential immune relationship between SLE and healthy samples by GSEA to explore whether there are immune differences between the two. The immune gene sets were downloaded from the ImmuneSigDB subset of MSigDB (http://www.gsea-msigdb.org/gsea/msigdb/genesets.jsp?collection=IMMUNESIGDB).

### Feature selection

We performed the least absolute shrinkage and selection operation (Lasso) regression using the glmnet package. Lasso regression provides a new feature selection algorithm that can solve the collinearity problem well and screen out representative variable features. The recursive feature elimination (RFE) method combined with a random forest classifier was used for feature selection using the caret package and a 10-fold cross-validation was performed. Immediately after, the importance scores of the random forest classifier for the feature genes were also performed using the caret package and the randomForest package. Firstly, a grid search method was used to find the best parameter mtry for fitting the random forest dataset. Secondly, the 10-fold cross-validation with five repetitions and multiple training rounds was performed. Finally, the signature genes’ importance score was performed, and the genes with score >10 were marked as the most important genes we need. The mtry parameter is the number of variables randomly sampled when constructing decision tree branches in random forest modeling. Choosing the appropriate value of mtry can reduce the prediction error rate of the random forest model and thus improve the performance of the model.

### Random forest for building a disease risk prediction model for SLE

To begin with, we incorporated the signature genes screened in the feature selection into the disease risk prediction model for SLE with random forest. Next, a grid search method using the caret package and randomForest package was used to determine the best parameter mtry for a good random forest fitted dataset. Thirdly, a 10-fold cross-validation with five iterations and multiple training rounds was performed to optimize the model and reduce overfitting based on accuracy. Ultimately, the SLE random forest diagnostic model with optimal parameters mtry was constructed and applied to the training dataset for 10-fold cross-validation to determine model robustness. The accuracy of the results was calculated by the confusionMatrix function. Using the pROC package, we calculated the area under the receiver operator characteristic (ROC) curve (AUC).

### Verification using validation datasets

On the validation dataset and the external validation dataset (GSE99967 and GSE185047), the efficacy of the disease risk prediction model for SLE with random forest was confirmed. For batch effects between GSE99967 (**Supplement Figure S1B**) and the training dataset as well as between GSE185047 (**Supplement Figure S1C**) and the training dataset, we once more utilized the Combat function to adjust before running the model testing. To further demonstrate the validity of the developed model, we determined the optimal parameter mtry, and a 10-fold cross-validation of the optimal disease risk prediction model for SLE with random forest using the same signature genes in the context of high-throughput sequencing. The AUC was calculated using the pROC package. The accuracy was estimated using the confusionMatrix function.

### Statistical analysis

All statistical analyses were performed with R software (version 4.1.3). P < 0.05 was considered statistically significant.

## Results

### Study design

**Figure 1:**
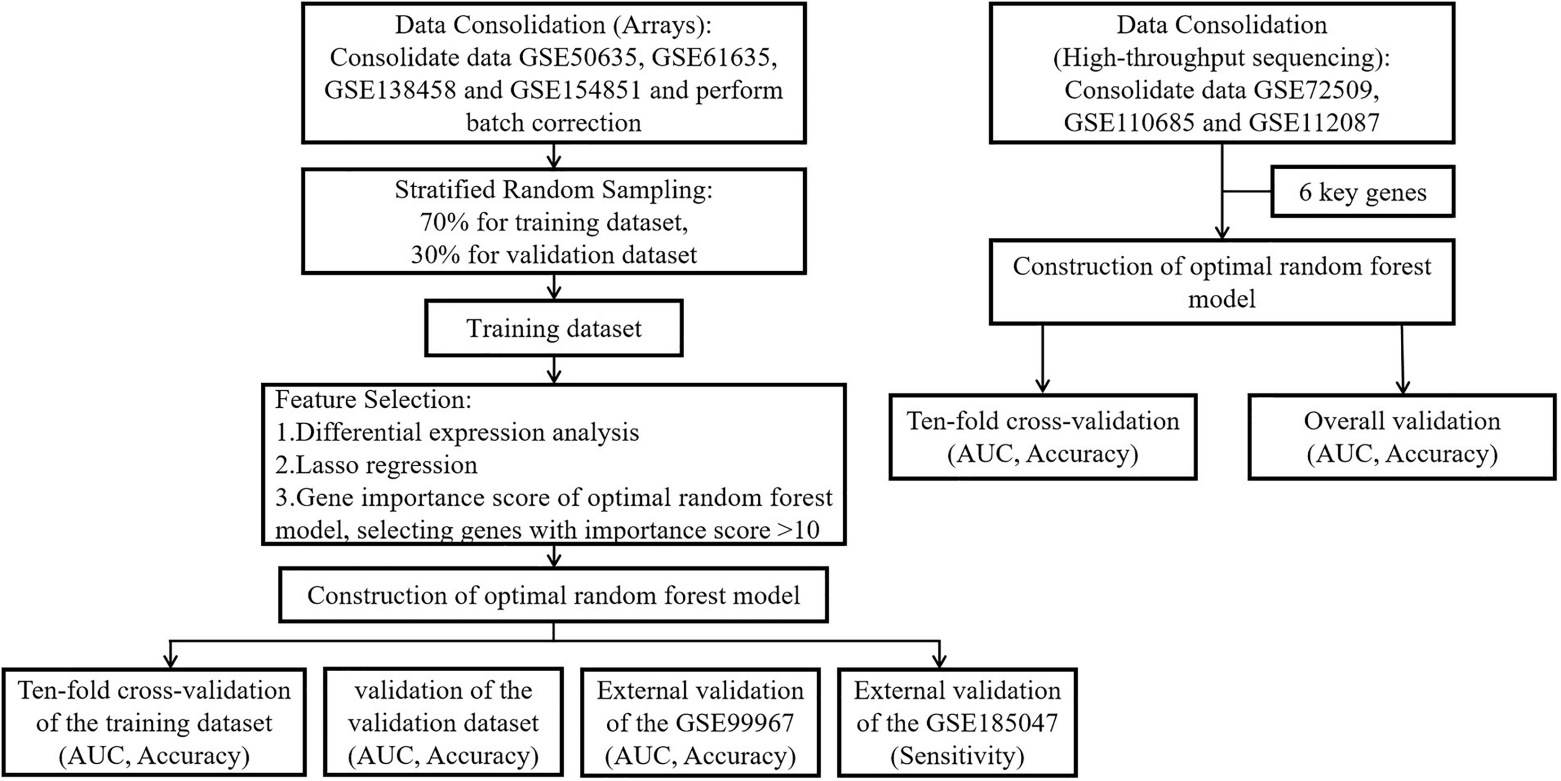
The flow chart of this study. Step 1: We merged the GSE50635, GSE61635, GSE138458 and GSE154851 datasets into a large dataset. Step 2: Stratified random sampling methods were used by us for the partitioning of large data sets, and the ratio of the training dataset to the validation dataset was 7:3. Step 3: We performed differential expression analysis, Lasso regression, RF-RFE and feature importance score of RF in the training dataset to screen key genes. Step 4: A random forest prediction model was constructed through the inclusion of key genes. Step 5: We used 10-fold cross-validation to check the robustness of the training dataset and validated the model using the validation dataset and two external validation datasets to obtain the AUC, accuracy, and sensitivity. Step 6: In high-throughput sequencing (GSE72509, GSE110685 and GSE112087), we directly incorporated key genes using 10-fold cross-validation to demonstrate that the random forest prediction model is equally well robust in the context of high-throughput sequencing.

**Figure 1** depicts the entire study flow.

### Identification of DEGs

**Figure 2:**
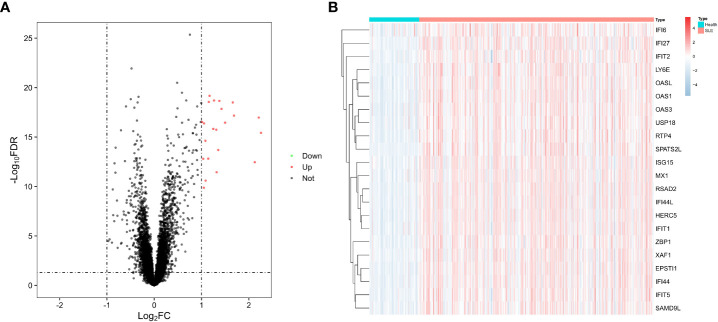
Differential genes. **(A)** Volcano diagram with 22 genes with significant differences, red dots indicate up-regulated genes, black dots indicate non-differentiated genes, and green dots indicate down-regulated genes. **(B)** Heat map of 22 differential genes with upregulation trends.

We performed a stratified random sampling of the data and divided it into a training dataset (70%) and a validation dataset (30%), where the SLE samples and healthy samples of the training dataset were 334 and 71, respectively, and the SLE samples and healthy samples of the validation dataset were 143 and 30, respectively. Then, Differential expression analysis was performed on the training dataset for the DEGs screening, and 22 significant DEGs associated with SLE were finally identified based on significance criteria. A volcano plot was used to depict the expression status of all DEGs (**Figure 2A**). We found that the expression trends of all DEGs were up-regulated. Through the heat map, we could see that the expression levels of DEGs in SLE were all trended up-regulated and significantly different compared to the control group (**Figure 2B**).

### Enrichment analysis

**Figure 3:**
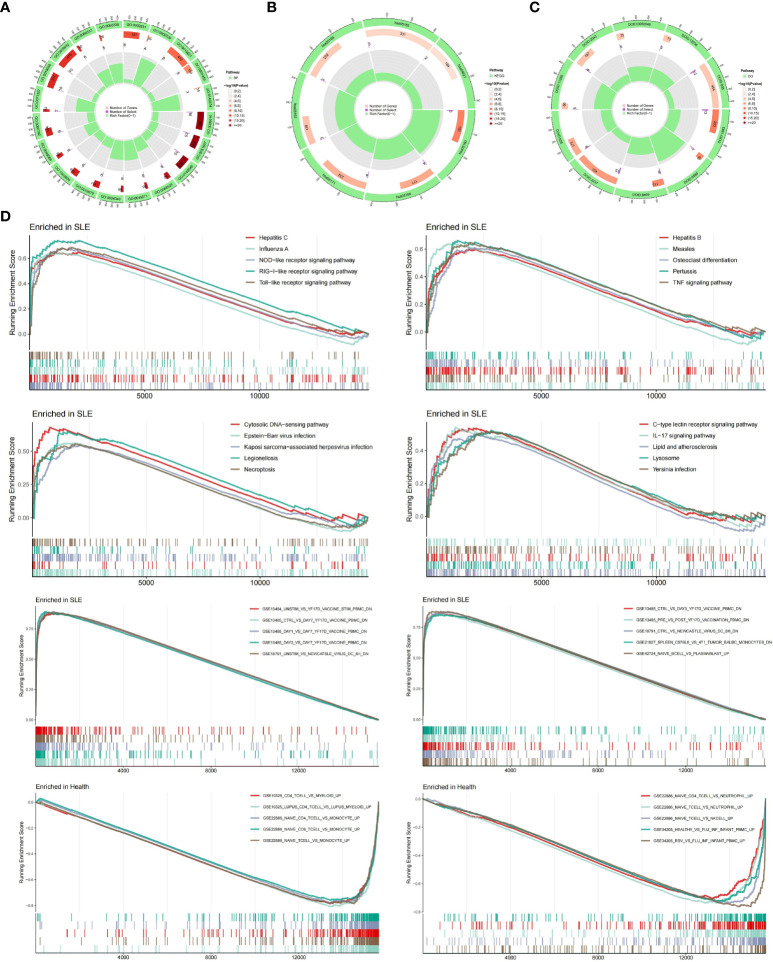
Enrichment Analysis. **(A)** Ring diagram of biological processes analyzed by GO enrichment. **(B)** Ring diagram of KEGG enrichment analysis. **(C)** Ring diagram of DO enrichment analysis. **(D)** About pathway-related and immune-related GSEA.

We performed the GO, KEGG and DO enrichment analysis of 22 DEGs. In terms of biological processes (**Figure 3A**), the results showed that DEGs were significantly enriched in response to viral responses and in response to type I interferon. According to KEGG analysis (**Figure 3B**), the results showed a major enrichment in viral-related disease pathways as well as nod-like receptor signaling pathways. In terms of DO analysis (**Figure 3C**), it suggested that important genes associated with SLE were also closely associated with inflammatory diseases such as hepatitis, encephalitis and influenza. Subsequently, the results of GSEA pathway differences (**Figure 3D**) showed that SLE patients cluster many inflammatory signaling pathways as well as inflammation-related diseases. Among them, lipids and atherosclerosis were positively associated with SLE. Intriguingly, we also found that necrotizing apoptosis also showed an up-regulation trend in SLE patients. The results of GSEA immune differences (**Figure 3D**) showed that SLE patients were enriched with many datasets of reduced peripheral blood mononuclear cells, while T lymphocytes showed a negative association with SLE patients.

### Screening for key genes

**Figure 4:**
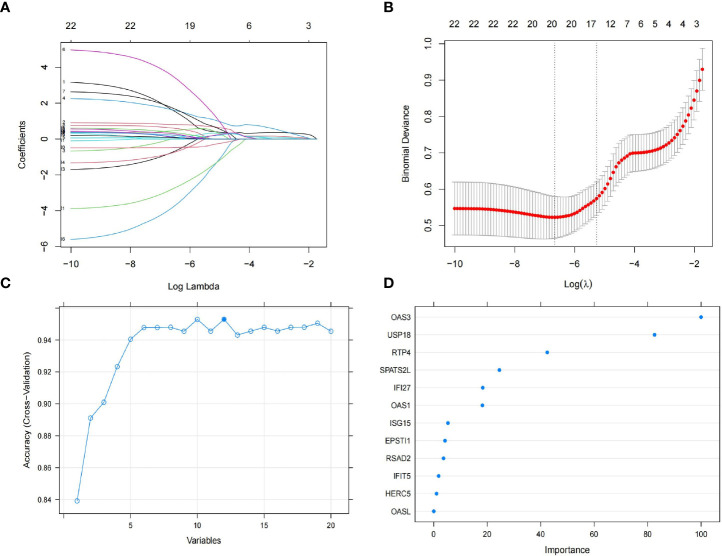
Feature selection. **(A)** The lasso regression curve of 22 DEGs. **(B)** The 10-fold cross-validation parameter (λ) options. **(C)** The 10-fold cross-validation of RMSE of signature gene combination of RF-RFE. **(D)** Gene importance scores for random forests.

To obtain the key genes, first, we entered 22 DEGs into the Lasso regression and performed a 10-fold cross-validation. Based on Lambda at minimum binomial deviation as a criterion (**Figures 4A, B**), we identified 20 candidate genes by compressing the feature variables. Second, we performed the feature selection of RF-RFE, and as shown in **Figure 4C**, it is clear that the model has the highest accuracy in the condition of 12 candidate genes. Third, we obtained the best model by incorporating 12 candidate genes into the random forest classifier and repeating the 10-fold cross-validation five times. To reduce the number of feature variables and still with good predictive power, we performed further analysis by identifying six genes with importance score> 10 as the final key genes. As shown in **Figure 4D**, OAS3 was the most important gene, followed by USP18, RTP4, SPATS2L, IFI27 and OAS1.

### Construction of the random forest model

**Figure 5:**
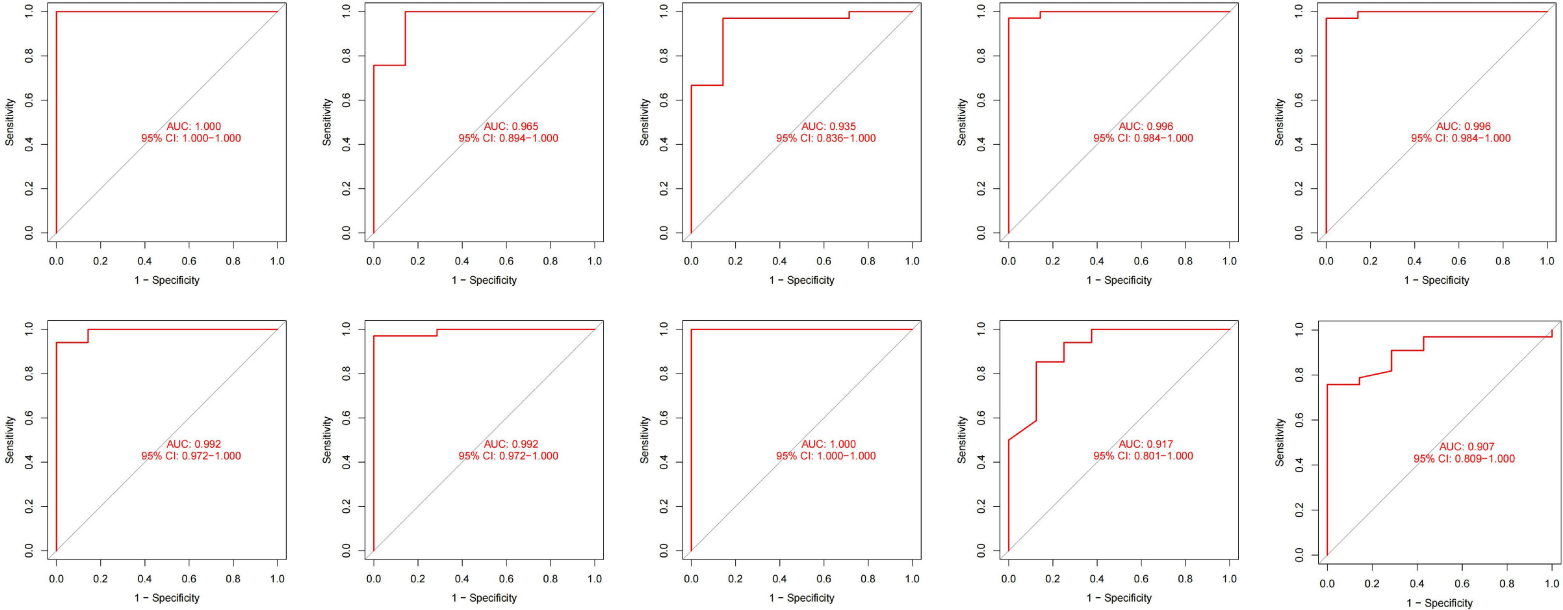
The 10-fold cross-validation verifies ROC curve results.

**Figure 6:**
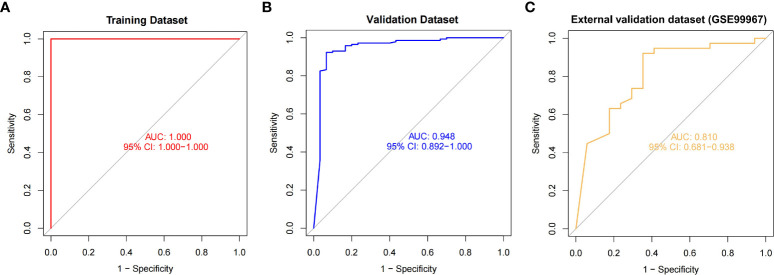
The ROC curves and their respective AUC values were used to evaluate the performance of the random forest model on the training **(A)**, validation **(B)** and external validation **(C)** datasets.

We incorporated OAS3, USP18, RTP4, SPATS2L, IFI27, and OAS1 into the random forest classifier. To optimize the performance of the model, we performed a grid search of the mtry parameters as well as calculated the model accuracy for each mtry using repeated 5 times 10-fold cross-validation. Finally, we locked the highest accuracy of the random forest disease risk prediction model when mtry was 3 and obtained the optimal random forest disease risk prediction model. Immediately after, we performed a robustness test on the model with a 10-fold cross-validation, and each result was represented by a ROC curve (**Figure 5**), while results for each accuracy have been shown in **Table 2**. The fact that the average AUC of the 10-fold cross-validation results exceeds 0.95 proved the reliability of the model. Finally, we estimated the AUC and accuracy for the whole training dataset, and the result was an AUC of 1 and its accuracy of 1 (**Figure 6A**).

**Table 2:** The 10-fold cross-validation results.

	Accuracy	AUC
Cross-validation 1	1.000	1.000
Cross-validation 2	0.975	0.965
Cross-validation 3	0.925	0.935
Cross-validation 4	0.976	0.996
Cross-validation 5	0.975	0.996
Cross-validation 6	0.976	0.992
Cross-validation 7	0.976	0.992
Cross-validation 8	1.000	1.000
Cross-validation 9	0.929	0.917
Cross-validation 10	0.875	0.907

### Internal validation of the random forest model

In the validation dataset, the AUC value estimated by ROC curve analysis was 0.948 and the accuracy estimated by confusion matrix was 0.9306, indicating the robustness of the model in identifying SLE (**Figure 6B**). These results suggested that we successfully developed a disease risk prediction model for SLE based on differential gene expression between SLE and normal samples. Additionally, we created model of OAS3 and model without OAS3, and their respective AUCs were 0.822 and 0.926 (**Supplement Figure S2**). We discovered that the addition of the most important gene, OAS3, boosted rather than diminished the model performance for the models built based on the other five genes based on the AUC values of the prior validation set of six gene disease risk prediction model.

### External validation of the random forest model

We further performed model validation using an external validation dataset (GSE99967). The result of the ROC curve analysis shows (**Figure 6C**) that the AUC value is 0.810, which still has high differential diagnostic power. Using the confusion matrix, we found an accuracy of 0.836 and a sensitivity of 0.921. The above sensitivity illustrates the better sensitivity of the model in patients suffering from SLE. Thus, we extracted the gene expression profiles of 87 SLE patients from another external validation dataset (GSE185047). By incorporating the data into the model, we found that 83 SLEs were detected in 87 SLE patients, with a sensitivity of 0.954.

### Application of random forest model in high-throughput sequencing

**Figure 7:**
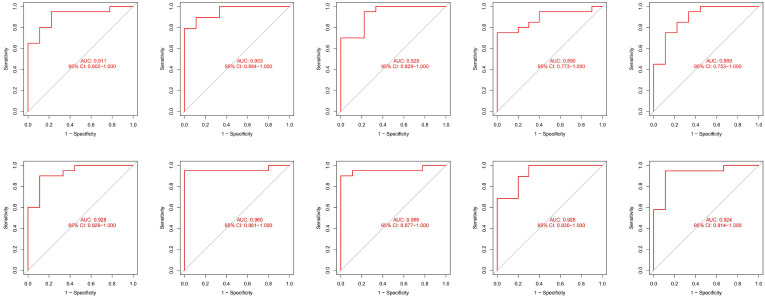
The ROC curve results were verified by 10-fold cross-validation under high-throughput conditions.

Due to the slight differences between high-throughput sequencing and array expression matrix, we separate high-throughput sequencing from array expression matrix and construct a disease risk prediction model for SLE in the context of high-throughput sequencing. We still used OAS3, USP18, RTP4, SPATS2L, IFI27 and OAS1 to incorporate the random forest classifier. The optimal model was filtered using repeated 5 times 10-fold cross-validation for grid search of mtry, and the best model was finally obtained when mtry was 3. Model robustness was also demonstrated using 10-fold cross-validation, as was done for the array expression matrix. Each result was represented by a ROC curve (**Figure 7**), and results for each accuracy have been shown in **Table 3**. The fact that the average AUC of the 10-fold cross-validation results exceeds 0.90 again demonstrated that the model was still reliable for high-throughput sequencing.

**Table 3:** The 10-fold cross-validation results.

	Accuracy	AUC
Cross-validation 1	0.793	0.911
Cross-validation 2	0.857	0.953
Cross-validation 3	0.897	0.928
Cross-validation 4	0.800	0.890
Cross-validation 5	0.828	0.889
Cross-validation 6	0.862	0.928
Cross-validation 7	0.967	0.960
Cross-validation 8	0.897	0.956
Cross-validation 9	0.759	0.926
Cross-validation 10	0.857	0.924

## Discussion

SLE is a chronic autoimmune disease with limited treatment options. Effective prediction and identification is the key to improve the survival of SLE patients (16). However, the precise mechanism of SLE’s occurs remains unknown. Currently, the conventional diagnostic criteria for SLE are still based on clinical manifestations and serum autoantibodies (17), with SLE suspected based on clinical findings and then laboratory tests to support the diagnosis. Clinical diagnosis of SLE is rarely made promptly because symptoms overlap with those of other skin diseases and other autoimmune disorders. It is essential to identify biomarkers that have a strong correlation with SLE. Due to the advances in machine learning and public gene expression data, we are better able to infer biomarkers significantly associated with disease (18).

In our study, we built the disease risk prediction model for SLE on a random forest algorithm as a way to distinguish SLE patient blood from normal human blood. With the rapid development of bioinformatics, the classification evidence of diseases like SLE can be well supported by strong evidence. To identify the DEGs of SLE, we first combined four GEO datasets (GSE50635, GSE61635, GSE138458 and GSE154851) and performed stratified random sampling to divide the training dataset (70%) from the validation dataset (30%). GO, KEGG, DO, and GSEA enrichment analysis were then performed. According to GO and KEGG analysis, the DEGs were associated with a large number of biological processes and pathways which reflect the dynamics and complexity of the pathogenesis. Many studies have been conducted to support our findings, and previous studies have shown that type I interferon is a key pathway in the pathogenesis of SLE (19, 20). A recent study by Vital et al. (21) found that anifrolumab, a type I interferon (IFN) receptor antagonist, showed the therapeutic benefit of anifrolumab in patients with SLE in patients with high interferon gene profile with greater baseline disease activity and abnormal serologic markers. Biswas et al. (22) found that type I interferon and Th17 pathways coexist and jointly regulate the pathogenesis of SLE. In a recent study, Caielli et al. (23) reported that mitochondrial dysfunction contributes to SLE pathogenesis, where the mechanism is a defect in the autophagic removal of the mitochondrial pathway during erythroid maturation, leading to the accumulation of red blood cells carrying mitochondria in SLE patients and the induction of IFN production through the activation of cGAS in macrophages. GSEA analysis is superior to KEGG analysis and provides a better understanding of the internal changes in the organism. The aggregation of many inflammatory signaling pathways and inflammation-related diseases in SLE patients indicates that the pathogenesis of SLE patients is associated with autoimmune abnormalities, which has been the consensus of those studying SLE. Among them, lipids and atherosclerosis were positively correlated with SLE, which suggests that SLE patients are prone to atherosclerosis, consistent with the SLE Complications Study (24, 25), and the development of atherosclerosis is closely related to immune inflammation. We found that necroptosis, a cysteine-independent form of programmed necrotic cell death, is upregulated in SLE patients and that necroptosis is closely associated with immune inflammation (26), with the pathway-essential RIPK3 promoting NLRP3 inflammasome activation and IL-1 β inflammatory response (27) as a way to induce and amplify inflammatory responses. Recently, it has also been suggested that necroptosis may be involved in the pathogenesis and development of SLE and that elevated IFN signaling in SLE increases necroptosis, which leads to tissue damage (28). Nonetheless, necroptosis remains poorly studied in SLE, and its role in the pathogenesis and development of SLE still needs further exploration.

Further performance of the RF classifier importance score screened for 6 key genes, namely OAS3, USP18, RTP4, SPATS2L, IFI27 and OAS1. Previous studies support our findings. 2’-5’-oligoadenylate synthetase 3 (OAS3), one of the genes encoding interferon-inducible antiviral enzymes, plays a key role in antiviral action and signal transduction. Ubiquitin specific peptidase 18 (USP18) is a member of the ubiquitin-specific protease (UBP) family of enzymes. This gene is not only physiologically relevant, but has also been implicated in the pathogenesis of various human diseases, including infectious diseases, neurological disorders, and cancer (29). It is strongly induced by type I IFN but is also able to negatively feedback inhibit type I IFN signaling (30, 31). Receptor transporter protein 4 (RTP4) is associated with lupus nephritis (32). It has been shown that type I IFN induces RTP4 and binds to the TANK-binding kinase (TBK1) complex, interfering with the expression of TBK1 and IFN regulatory factor 3 (33). Spermatogenesis associated serine rich 2 like (SPATS2L) has been poorly studied in SLE, but we were able to find, based on previous studies, that SPATS2L has an important role in the development of asthma (34) and is closely associated with the prognosis of glioma patients (35). Interferon Alpha Inducible Protein 27 (IFI27) is involved in type I interferon-induced apoptosis (36) and may be a potential diagnostic marker for SLE as well as an immunotherapeutic target (37). 2’-5’-oligoadenylate synthetase 1 (OAS1) is a type I interferon-inducible gene that plays a key role in the innate cellular antiviral response and is associated with other cellular processes such as cell growth and apoptosis, and is an SLE diagnostic biomarker. Although the screened genes were all reported in SLE, this can illustrate the reliability of machine learning to screen key genes.

The highlights of our study are the innovative combination of Lasso and RF methods and the excellent results produced in terms of predictive power. The feature selection method of Lasso (38–41) and RF (42–45) has been widely used in biology as a way to better identify key biomarkers. Before that, there is still no study to construct a prediction model for SLE based on gene sequencing, especially since our study is based on whole blood samples from SLE patients, which are easy to obtain and manipulate. On the other hand, due to the high-speed advances of sequencing and the difference between array expression matrix and high-throughput sequencing, we constructed the disease risk prediction model for SLE separately as a way to accommodate the difference of different sequencing.

The AUCs of our model on the training dataset, validation dataset, and external validation dataset (GSE99967) are 1, 0.948, and 0.810, respectively, in the matrix expression of the array, indicating the strong robust of our model. In addition, our model demonstrated a high SLE sensitivity of 0.954 in the external validation dataset (GSE185047) and a sensitivity of up to 0.921 in GSE99967, showing a very high sensitivity of the model to SLE. In the high-throughput sequencing datasets, the average AUC of all 10 cross-validations is above 0.9, which also indicates that our model is very robust and well suited for Identification and diagnosis of SLE.

Using the gene transcriptome level, we investigated the validity and reliability of machine learning in the disease risk prediction of SLE. As a result, we were able to successfully construct a novel disease risk prediction model for SLE that can be utilized as a novel SLE disease risk prediction tool and help to identify SLE.

Even so, there are some limitations to our study. 1) Many of these public data do not contain detailed clinical data on patients and health samples, thus lacking attention to the different kinds of SLE and medication use. 2) Although we merged as many datasets as possible into a larger dataset to build the model, it still falls short of the number of data samples needed for machine learning. If conditions permit, we can include more research data in the training dataset in the future. 3) The overfitting of the model construction is objective and difficult to eliminate, but we use 10-fold cross-validation in the modeling process to minimize the overfitting problem. Checking for overfitting is not a complete solution, but it is still very helpful. However, this means that even if we get good model results on the validation dataset, there is no shortage of different data with noise in reality, and the actual generalization ability may not be good. 4) The model has not been tested in practical applications to predict SLE patients. Thus, we still need more research data in the future to test the robustness and generalization ability of the model.

## Conclusions

In conclusion, in our thorough examination of the SLE dataset in the GEO database, we found that the key biomarkers OAS3, USP18, RTP4, SPATS2L, IFI27 and OAS1, which were significantly associated with SLE, were able to jointly construct the disease risk prediction model for SLE with random forest. And for the first time, we used random forest machine learning algorithms to create a strong prediction model based on six genes to predict SLE.

## Data availability statement

The datasets presented in this study can be found in online repositories. The names of the repository/repositories and accession number(s) can be found in the article/**Supplementary Material**.

## Author contributions

XL and SM contributed to the conception and design of the study. HC and LH drafted the manuscript and collected and analyzed the data. XJ, YW, and YB were responsible for data curation and validation. All authors contributed to the article and approved the submitted version.

## Funding

This study was supported by NSFC grants (Nos. 81773363, 81872558 and 81972969); The key R & D project of the Department of Science and Technology of Zhejiang Province (2020C03028), the key project jointly built by the Ministry of Zhejiang Health Commission (2021438235), and Major Project of Wenzhou Bureau of Science and Technology (2020ZY0011).

## Conflict of interest

The authors declare that the research was conducted in the absence of any commercial or financial relationships that could be construed as a potential conflict of interest.

## Publisher’s note

All claims expressed in this article are solely those of the authors and do not necessarily represent those of their affiliated organizations, or those of the publisher, the editors and the reviewers. Any product that may be evaluated in this article, or claim that may be made by its manufacturer, is not guaranteed or endorsed by the publisher.

## Supplementary material

Supplementary Figure 1 Box plot of gene distribution before and after batch effect correction. **(A)** Batch correction for GSE138458, GSE154851, GSE50635, and GSE61635. **(B)** Batch correction for Training dataset and GSE99967. **(C)** Batch correction for Training dataset and GSE185047.

Supplementary Figure 2 ROC curves for the model of OAS3 **(A)** and the model without OAS3 **(B)** in the validation dataset.

## Abbreviations

AUC, area under the curve; BP, biological processes; DEGs, differentially expressed genes; DO, Disease Ontology; FDR, false discovery rate; GEO, Gene Expression Omnibus; GO, Gene Ontology; GSEA, Gene Set Enrichment Analysis; IFN, Interferon; KEGG, Kyoto Encyclopedia of Genes and Genomes; Log2FC, log2fold change; NCBI, National Center for Biotechnology Information; RF, random forest; ROC, receiver operator characteristic; RFE, recursive feature elimination; SLE, Systemic lupus erythematosus.
