# Supplementary figures and images for: Establishment and analysis of a disease risk prediction model for the systemic lupus erythematosus with random forest
Source: Front Immunol. 2022 Nov 1;13:1025688. doi: 10.3389/fimmu.2022.1025688 (PMC9667742; doi:10.3389/fimmu.2022.1025688)

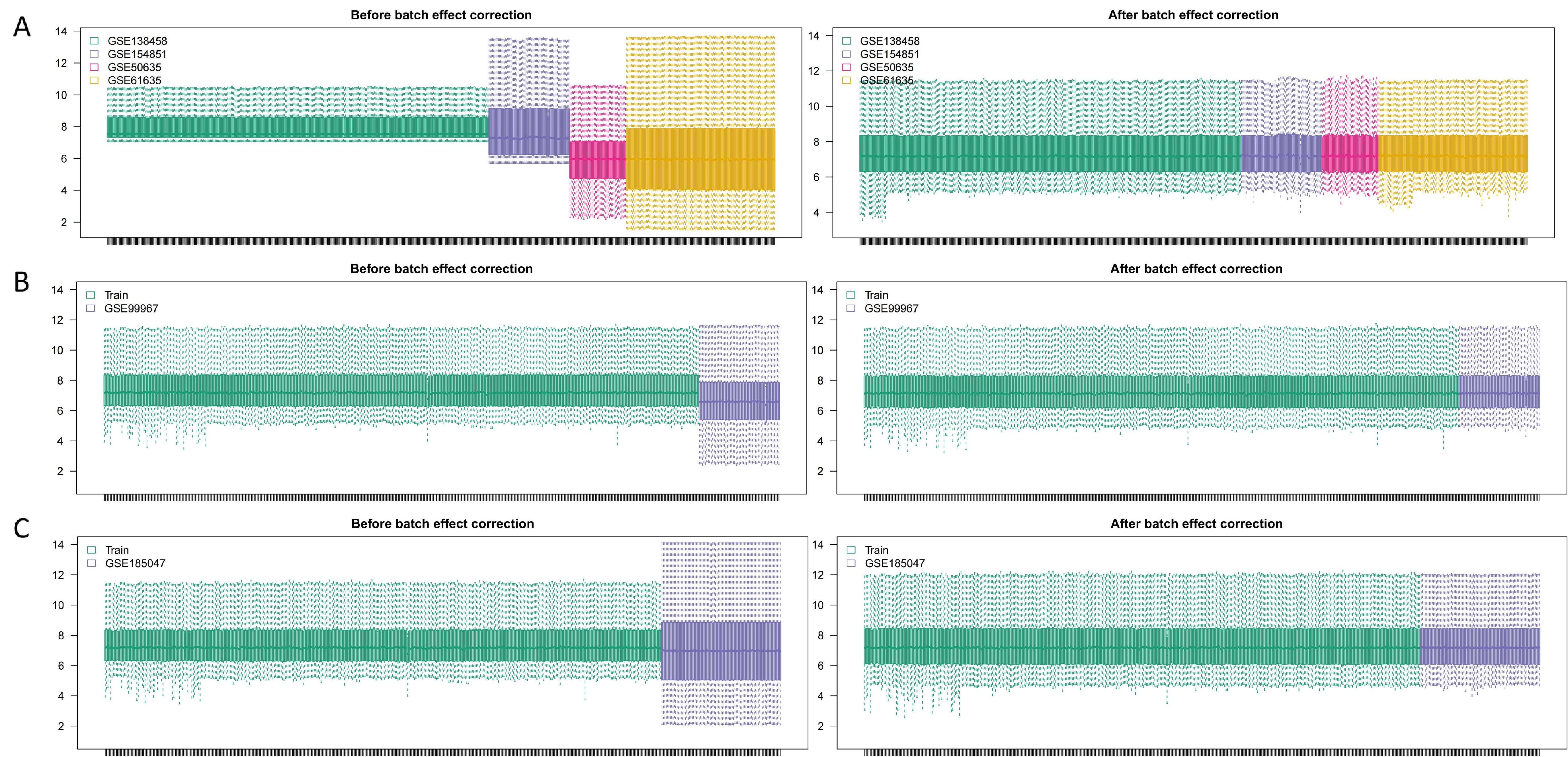

Supplement: Supplementary Figure 1 — Box plot of gene distribution before and after batch effect correction. (A) Batch correction for GSE138458, GSE154851, GSE50635, and GSE61635. (B) Batch correction for Training dataset and GSE99967. (C) Batch correction for Training dataset and GSE185047. [file Image_1.jpeg]

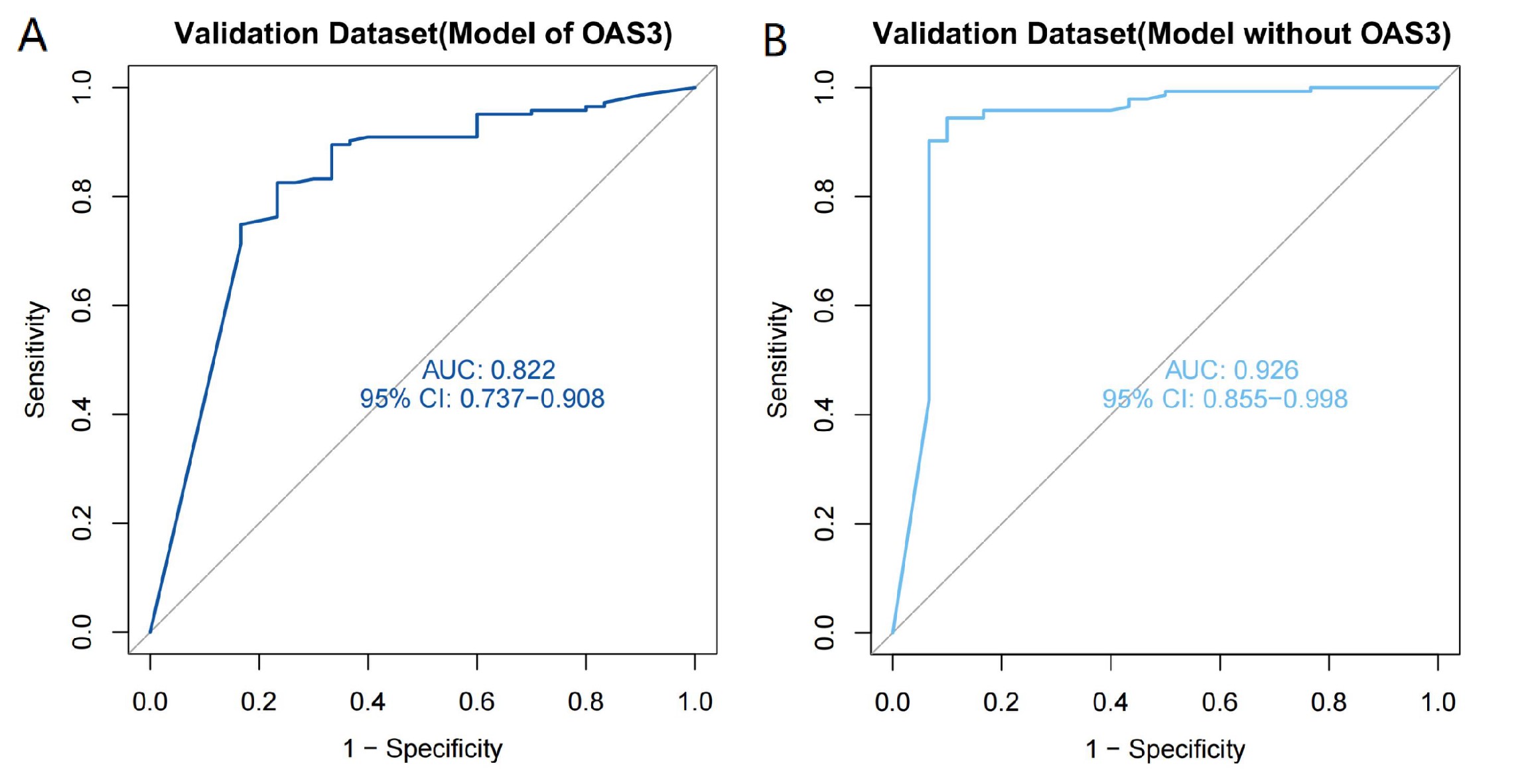

Supplement: Supplementary Figure 2 — ROC curves for the model of OAS3 (A) and the model without OAS3 (B) in the validation dataset. [file Image_2.jpeg]
